# Supplementary material for: Vegetation feedback causes delayed ecosystem response to East Asian Summer Monsoon Rainfall during the Holocene
Source: Nat Commun. 2021 Mar 23;12:1843. doi: 10.1038/s41467-021-22087-2 (PMC7988120; doi:10.1038/s41467-021-22087-2)
Supplement: Supplementary file 1 — Supplementary Information [file 41467_2021_22087_MOESM1_ESM.pdf]

Supplementary Information for

Vegetation Feedback causes Delayed Ecosystem Response to East Asian Summer Monsoon Rainfall during the Holocene

by Cheng *et al.*

This PDF file includes:

Supplementary Discussion
Supplementary Figures 1 to 8
Supplementary Tables 1 to 4
Supplementary Data 1
Supplementary References

Supplementary Discussion

1. AMOC impact on Holocene EASM in TraCE21. AMOC intensification from early to mid-Holocene has been suggested to strengthen EASM (*1, 2*). In our model, AMOC is indeed strengthened from 10 to 6 ka by the melt water flux (by 2 Sv) after the recovery of the 8.2ka event (Supplementary Fig. 7), which seems to contribute to a modest increase of EASM wind, rainfall and soil wetting in NC (green in Figs. 2, B-D). However, this AMOC impact is small compared to those from the orbital forcing and ice-sheet retreating (Fig. 2). The AMOC is also increased slightly (by 1 Sv) by the orbital forcing, but this secondary impact of the AMOC change (forced by the orbital forcing) on EASM, if any, is completely overwhelmed by the direct insolation forcing on EASM (red in Figs. 2, B-D).

2. Relationship between evaporation and soil moisture variation. Ground evaporation is usually considered as a dominant factor on the long-term changes of soil moisture. In the traditional view, a larger evaporation should correspond to a drier soil, and vice versus. However, model evaporation over NC peaks in the mid-Holocene (Fig. 3A), the same as the local soil moisture (Fig. 1F), which appears to be consistent with some studies on the evaporation-soil moisture relationship for semi-arid region in modern observations (*3*). Therefore, in our model, the change of evaporation appears to be the result, instead of the cause, of the changes of the soil moisture over NC.

3. Identification of vegetation mechanism in sensitivity experiments. Sensitivity experiments show that, over the NC region, the rainfall effect alone indeed leads to a drier soil, but has little change in vegetation (blue in Figs. 4, C&D). However, the change in thermal forcing leads to a large shift from grasses to trees, and a surface wetting (red in Figs. 4, C&D). Furthermore, the impact of thermal forcing is caused almost completely by the winter warming. This is shown in a further sensitivity experiment that use the anomalous insolation/temperature in the winter half year only (brown in Figs. 4, C&D, Exp.6 - Exp.1), which virtually reproduces the results of the year-round thermal forcing (red in Figs. 4, C&D). A separate experiment confirms that the summer thermal forcing plays little role (not shown). Physiologically, the winter warming crosses the cold threshold for broad leave trees and grasses (-17°C, *4*) and therefore favors the replacement from grasses to trees (Fig. 4A). With both the thermal forcing and rainfall combined, the model produces a surface wetting trend over NC (green in Figs. 4, C&D), qualitatively consistent with that in TraCE21 (Fig. 1G).

4. Choosing the number of analogs and the threshold. Paleoclimate reconstruction will be more reliable if the fossil sample has close modern analogues in the training-set. To find a suitable dissimilarity threshold (T), there is a trade-off between the reconstruction precision/accuracy and the utility for the majority of samples. T value (< 0.06) was

determined by the relatively low root mean squared error of prediction (RMSEP), high coefficient of determination (R^2) and over 98% of surface samples having analogues, through conducting a series of cross-validation experiments following Supplementary Ref. 5. Paleoclimate is reconstructed using the modern analogue technique (MAT), modern analogues of fossil samples are the closest surface samples measured by squared chord distance (SCD), and then in general the dissimilarity-weighted mean climate of the 3-7 closest modern analogues is assigned to fossil sample (*5*). In this study, we chose the 7 closest modern analogues based on the relationship between the number of modern analogues and the changes of R^2, RMSEP to maximize the precision and accuracy for paleoclimate reconstruction.

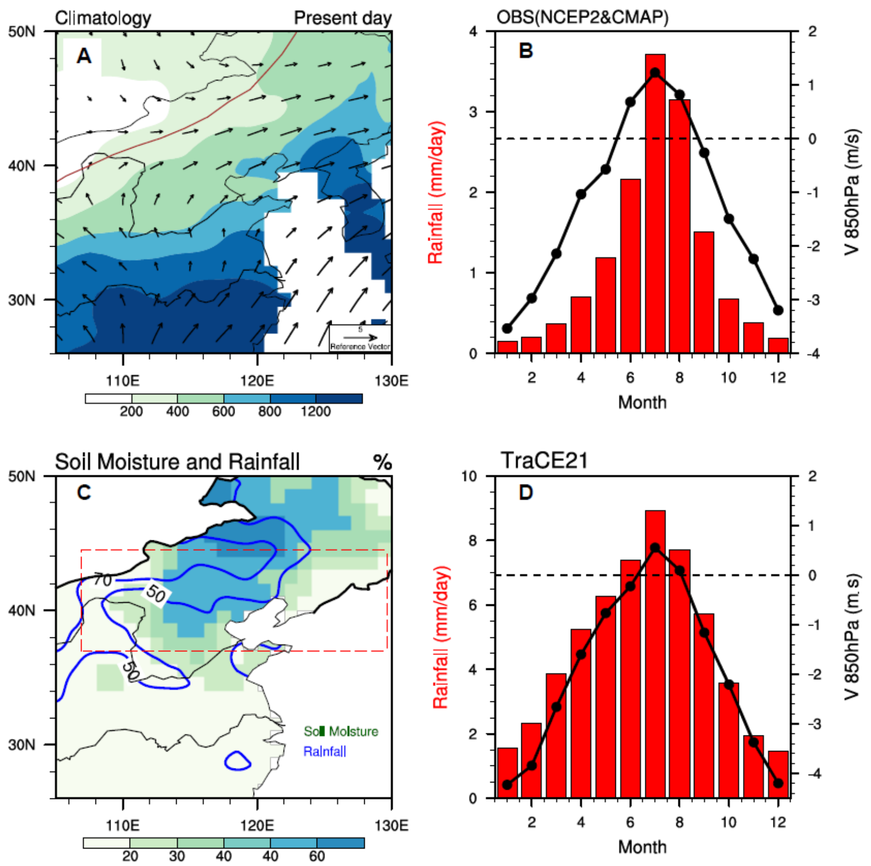

Supplementary Fig. 1. EASM climatology in the present observation and model. (A) Observed summer mean (JJA) 850 hPa wind (vectors, NCEP2) (m/s) and rainfall (shading, CMAP, *6*) (mm/year). The northern edge of the modern EASM (defined as the latitude of vanishing meridional wind v = 0) is indicated by the brown line. (B) Climatological seasonal cycles of rainfall and 850 hPa meridional wind at 850 over NC in the observation. The northerly monsoon wind arrives in the region in June, July and August in the EASM season, producing 65% of annual rainfall. (C) Magnitude of the relative change (%) of interannual variability of annual rainfall (contours) and soil moisture (shading). Red rectangle indicates the NC region (108°-129°E, 37°-45°N), the same as in Fig.1B, which is used for the average in b) here. (C) Same as (B) but for TraCE21 simulation at present, showing that the model reproduces the major feature of the seasonal cycle of wind and rainfall over NC.

Supplementary Table 1. Fossil pollen sites in Northern China used in this study.

No.	Site	Latitude (º)	Longitude (º)	Altitude (m)	Number of ^{14}C age	Reference
1	Gonghai	38.90	112.23	1860	18	Xu et al., 2017 (*7*)
2	Daihai99a	40.55	112.66	1221	8	Xiao et al., 2004 (*8*)

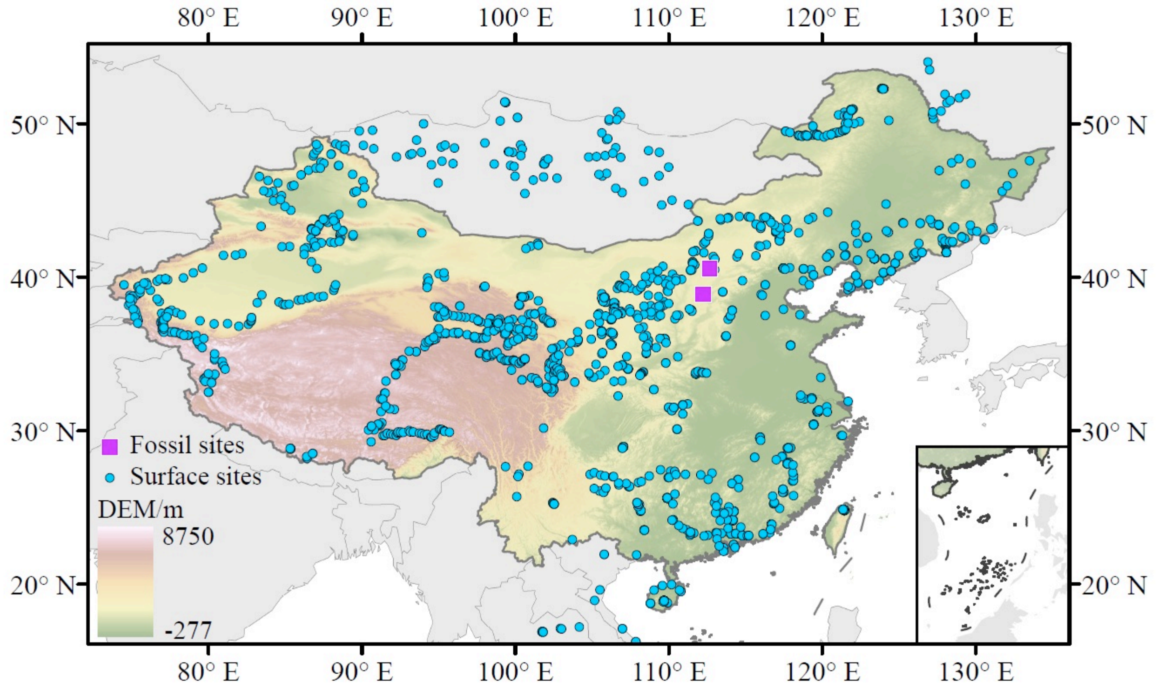

Supplementary Fig. 2. Pollen records. Sites (N=1865) of modern surface pollen samples (blue circle) used for paleoclimate reconstruction in the modern analogue technique (MAT). Sites (N=2) of fossil pollen are also marked (purple square) in NC. Shading is for topography (color). The information of the fossil pollen sites is given in Supplementary Table 1.

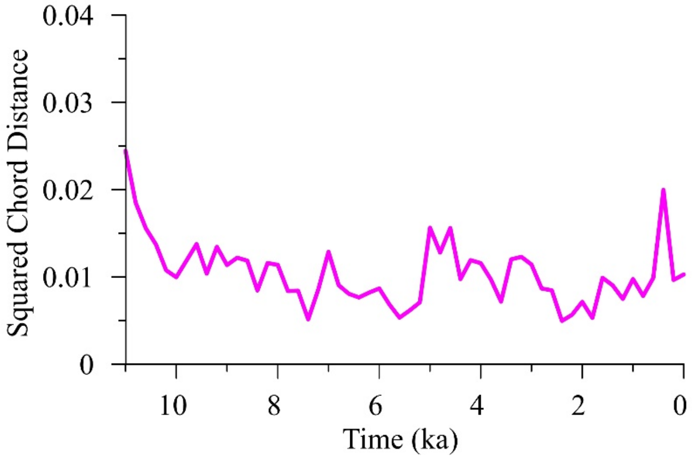

Supplementary Fig. 3. Squared chord distance between fossil samples and modern analogues during the Holocene for Daihai Lake.

Supplementary Table 2. Validation of reconstruction scheme within modern pollen datasets. Relationships between predicted values by the MAT method and observed climate values based on leave-one-out cross-validation analysis using the modern pollen datasets.

Climate variables	Correlation (R^2)	RMSE	No. Analogues	N
**ANNT**	0.77	3.7 ℃	7	1864
**ANNP**	0.91	186 mm	7	1864

ANNT: annual mean temperature, ANNP: annual mean precipitation.

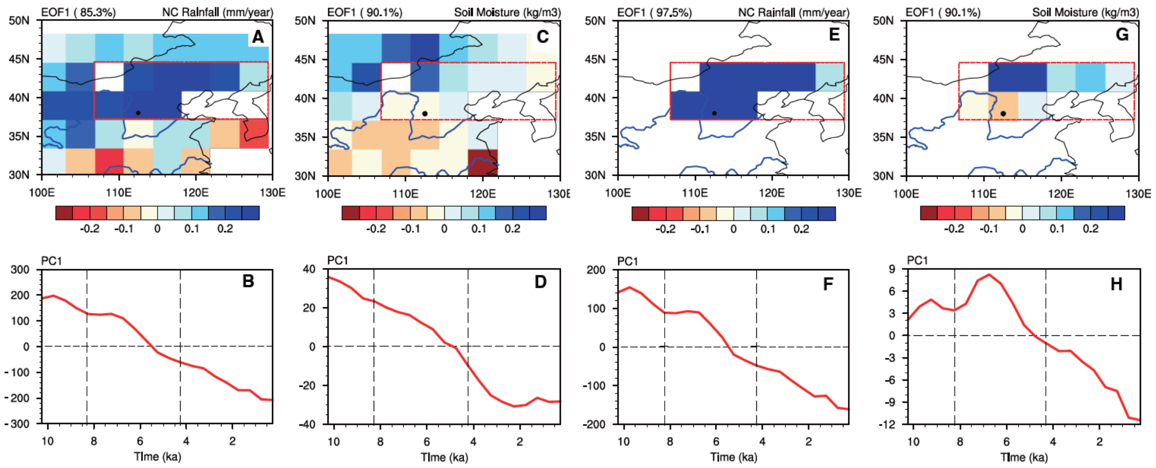

Supplementary Fig. 4. EOFs of rainfall and soil moisture over China in TraCE21. EOF1 and PC1 of annual rainfall ((A) and (B)) and upper layer (0.5-m) annual soil moisture ((C) and (D)) over the broad eastern and northern China (100-130°E, 30°-50°N). (E)-(H) are the same as (a)-(d) except for the NC region (108°-129°E, 37-45°N), as defined in Fig.1B. The black dot indicates the location of Gonghai Lake (*1*). Over the broad region of eastern and northern China, rainfall exhibits a dipole pattern, indicating a decrease from the early Holocene all the way to late Holocene over most of the northern part, but the opposite trend in the southern part along Yangzi-Huai River region. Soil moisture exhibits a similar monotonic change with a dipole pattern, but is shifted northward by ~5° to 10° in latitude relative to rainfall, likely caused by the stronger evaporation towards the south. The dominant dipole pattern of response suggests a spatial inhomogeneity of the EASM rainfall response in the Holocene, which has been suggested to be caused by the large scale monsoon moisture transport (*9*) and westerly jet migration (*10, 11*). In contrast, over the subdomain in the NC region, PC1 of soil moisture increases towards the mid-Holocene peak and then decreases towards late Holocene, with the southern most part experiencing a somewhat opposite change, although the PC1 of rainfall still exhibits a decreasing trend. This indicates that it may be difficult to make a robust reconstruction of rainfall and soil moisture over NC from a single site, especially in the southern part (*1*).

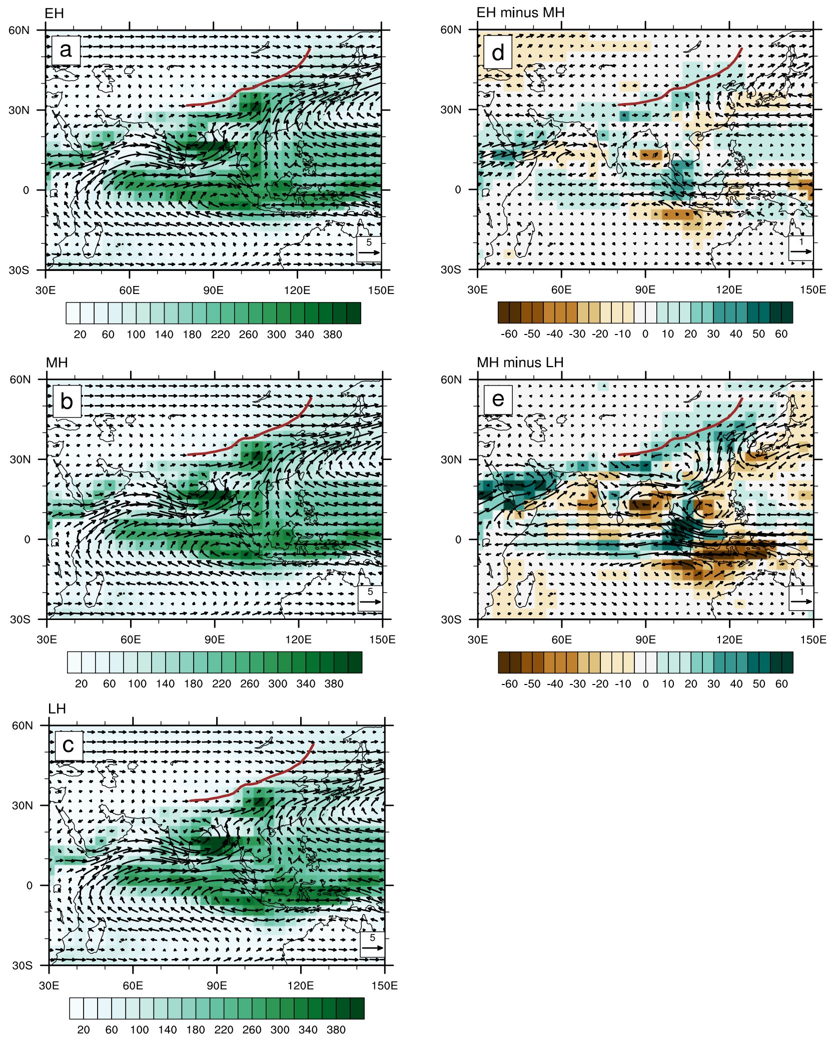

Supplementary Fig. 5. Vertically integrated vapor transport from surface to 500 hPa at early Holocene (EH, a), mid-Holocene (MH, b), late Holocene (LH, c), and its change from early to mid-Holocene (EH minus MH, d) and that from mid-Holocene to late Holocene (MH minus LH, e). The largely similar pattern of total moisture transport in (a) (b) and (c) suggests the climatological wind, and in turn moisture transport remains largely consistent in the Holocene, with the moisture source mainly from the Indian Ocean but also from the western North Pacific. Therefore, an increased rainfall in South Asia and Indian Ocean may help transport moisture into the NC region through the mean circulation advection on the anomalous moisture ($\bar{v}q'$). The difference of the moisture transport in (d) and (e), which are determined mainly by the changing wind and its advection of mean moisture ($\bar{q}v'$), shows that the NC rainfall may also be increased by the changing circulation transport of moisture, from the western North Pacific from mid- to early Holocene (in (d)) and from South Asia and Indian Ocean from late to mid-Holocene (in (e)).

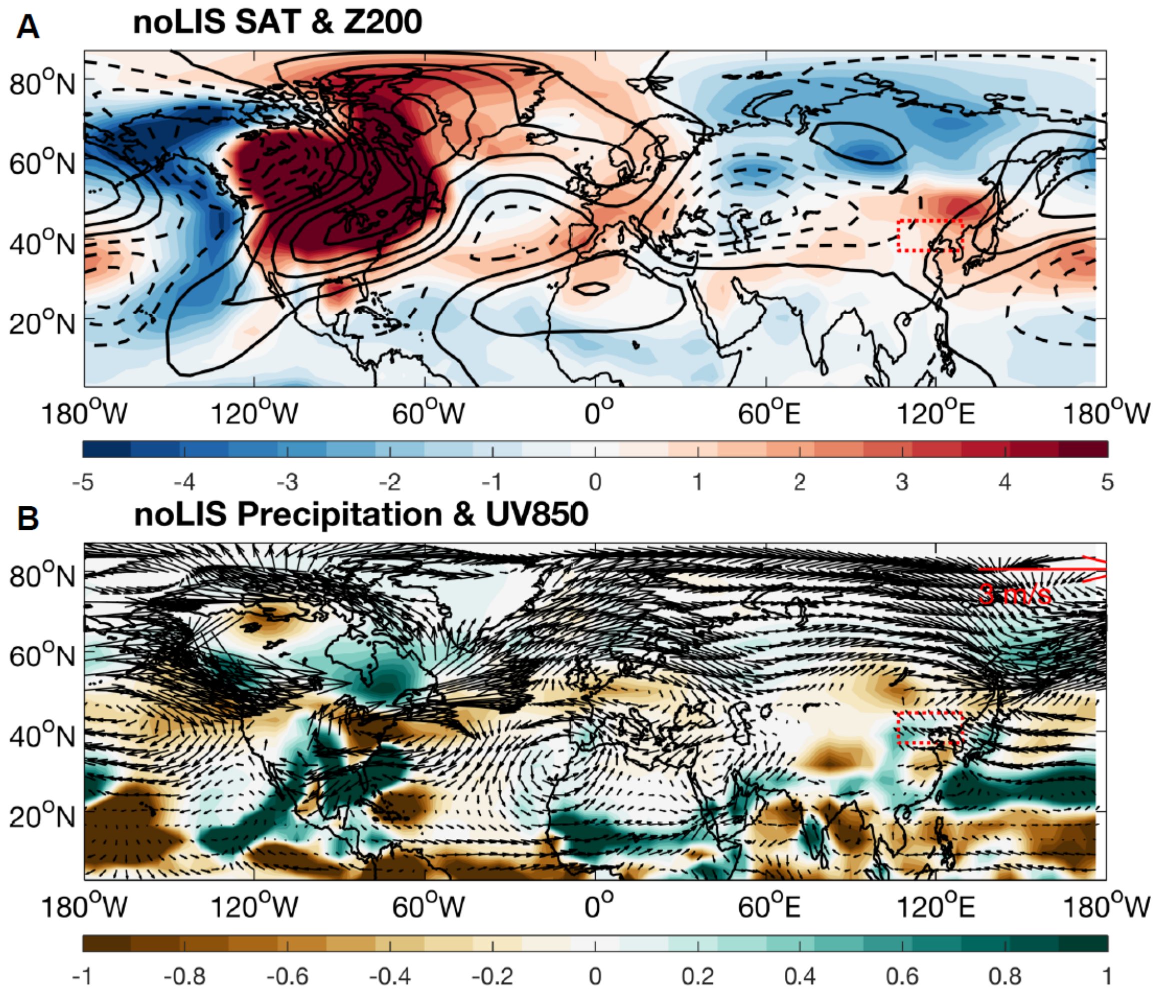

Supplementary Fig. 6. Atmospheric response to a lowering Laurentide Ice Sheet. Summer (JJA) atmospheric response of (A) SAT and 200 geopotential height and (B) 850 hPa wind and precipitation to a removal of Laurentide ice sheet in the atmospheric model CAM3 (the atmospheric component of CCSM3 used in TraCE21) coupled with a slab ocean. It is seen that the removal of ice sheet, as in the early Holocene, generates an atmospheric wave response of wave number 4, which propagates along the westerly wave-guide into the East Asia, enhancing the southerly monsoon wind and increasing rainfall over NC (but decreasing to the south). This is consistent with the ice sheet retreating effect that cancels the rainfall decline forced by the insolation in Fig.2C.

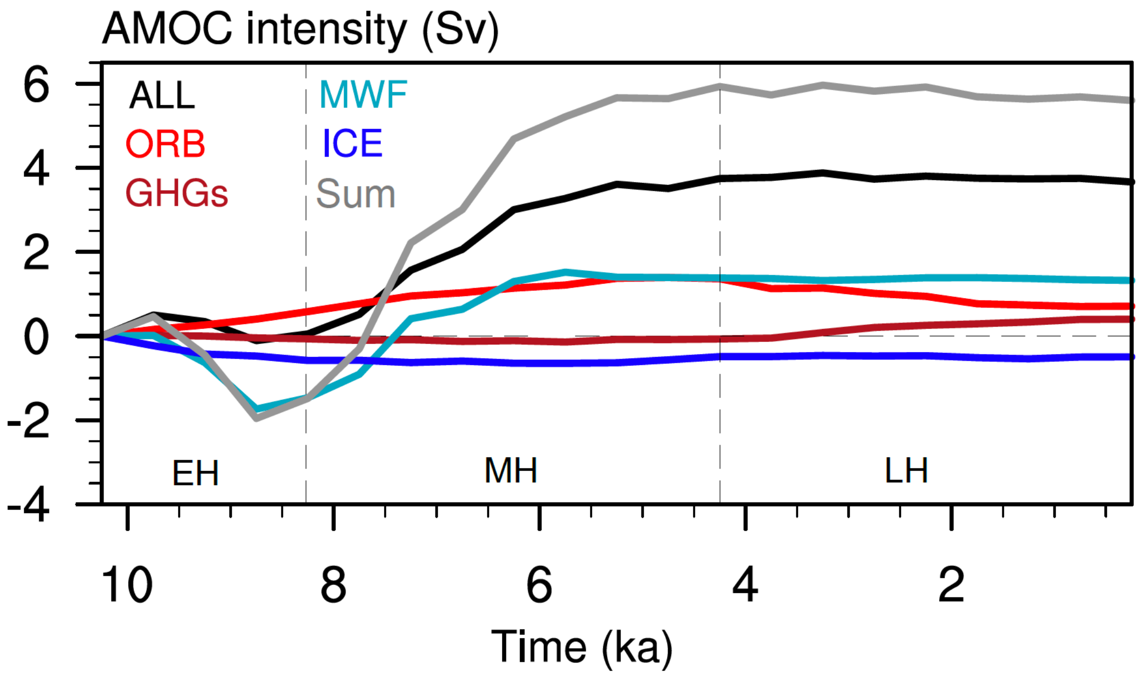

Supplementary Fig. 7. AMOC evolution in TraCE21 experiments. Time series of AMOC intensity (maximum transport below 500 m in the Atlantic) in the TraCE21 (All) experiment and four single forcing sensitivity experiments. All values are the anomalies from their values at 10.5ka. AMOC intensification from the early to mid- Holocene has been suggested to lead to a strengthening EASM (*1, 2*). In our model, AMOC is indeed strengthened by the orbital forcing (1 Sv) and melt water flux (4 Sv after 8.2ka event) over this period, and contributes to the strengthening of EASM and the wetting of NC soil (Figs. 2, B-D, green). However, these impacts of AMOC are small compared to those from the orbital forcing and ice-sheet retreating (Fig. 2).

Supplementary Table 3. Settings and results for land-vegetation coupled sensitivity experiments

Exp	Rainfall	Insolation/SAT	Wind/PS/Q	Tree (%)	Soil Moisture (kg/m^2)
1	9.5ka	9.5ka	9.5ka	0	293.83
2	6.5ka	6.5ka	6.5ka	95	313.48
3	6.5ka	6.5ka	9.5ka	95	309.77
4	6.5ka	9.5ka	9.5ka	4.2	292.38
5	9.5ka	6.5ka	9.5ka	95	310.98
6	9.5ka	6.5ka winter*	9.5ka	95	310.89

* Winter is defined as the average of the monthly output from November to the following March

Supplementary Table 4. Forcing for land-vegetation coupled sensitivity experiments derived from TraCE21.

	SATmin (℃)	SATmax (℃)	Solar radiation (W/m^2/year)	Rainfall (mm/year)	Q (g/kg)	PS (hPa)	WIND (m/s)
9.5ka	-18.9	20.25	2190	990	4.27	948.5	6.23
6.5ka	-15.3	20.3	2165	958	4.13	947.9	5.38

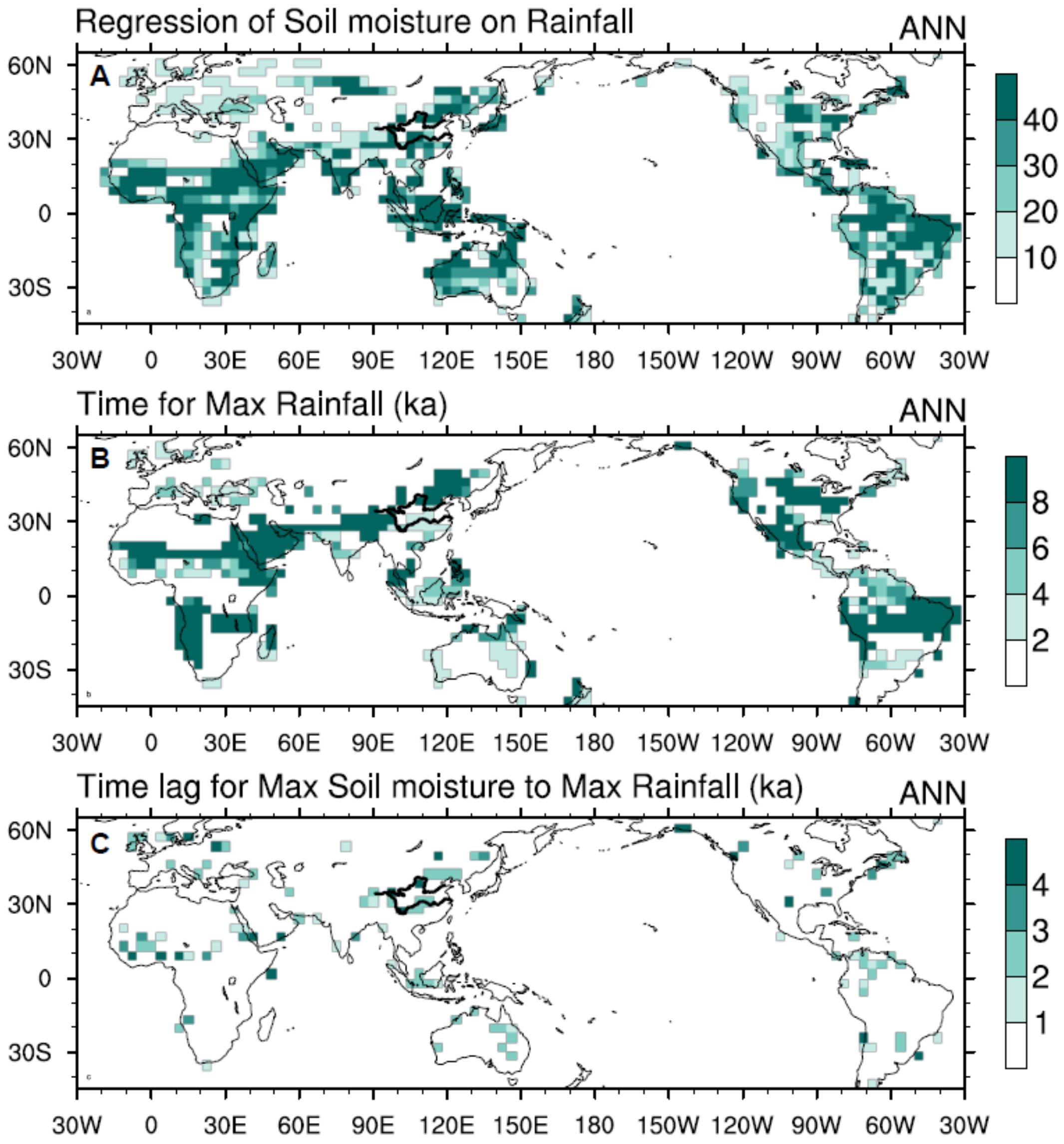

Supplementary Fig. 8. The delay of the environment to rainfall over the globe in TraCE21. (A) Regression of annual mean soil moisture on local annual mean rainfall (kg/m2 per mm/year). (B) Time (ka) of maximum rainfall over Holocene period. (C) Lags (ka) of the maximum soil moisture to peak local annual rainfall. The delayed response of the eco-environment to rainfall is seen to occur worldwide in some semi-arid regions of high climate sensitivity, although the mechanisms may not be exactly the same as in the NC region.

Supplementary Data 1. Source data used in Figs 1-4 including simulation result of TraCE and new reconstruction of annual rainfall in lakes of Daihai and Gonghai.

Supplementary References:

1. Chen, F., Xu, Q., Chen, J., Birks, H. J. B., Liu, J., Zhang, S., Jin, L., An, C., Telford, R. J., Cao, X., Wang, Z., Zhang, X., Selvaraj, K., Lu, H., Li, Y. C., Zheng, Z., Wang, H., Zhou, A., Dong, G., Zhang, J., Huang, X., Bloemendal, J. & Rao, Z. East Asian summer monsoon precipitation variability since the last deglaciation. *Sci. Rep.* **5**, 11186 (2015).

2. Wen, X., Liu, Z., Wang, S., Cheng, J. & Zhu, J. Correlation and anti-correlation of the East Asian summer and winter monsoons during the last 21,000 years. *Nat. Communi.* **7**, 11999 (2016).

3. Seneviratne, S. I., Corti, T., Davin, E. L., Hirschi, M., Jaeger, E. B., Lehner, I., Orlowsky, B. & Teuling, A. J. Investigating soil moisture-climate interactions in a changing climate: A review. *Earth-Sci. Rev.* **99**, 125-161 (2010).

4. Bonan, G. B., Levis, S., Sitch, S., Vertenstein, M. & Oleson, K. W. A dynamic global vegetation model for use with climate models: concepts and description of simulated vegetation dynamics. *Glob. Chang. Biol.* **9**, 1543-1566 (2003).

5. Williams, J. W. & Shuman, B. Obtaining accurate and precise environmental reconstructions from the modern analog technique and North American surface pollen dataset. *Quaternary Sci. Rev.* **27**, 669-687 (2008).

6. Xie, P. & Arkin, P. A. Global Precipitation: A 17-Year Monthly Analysis Based on Gauge Observations, Satellite Estimates, and Numerical Model Outputs. *Bull. Am. Meteorol. Soc.* **78**, 2539-2558 (1997).

7. Xu, Q., Chen, F., Zhang, S., Cao, X., Li, J., Li, Y., Li, M., Chen, J., Liu, J. & Wang, Z. Vegetation succession and East Asian Summer Monsoon Changes since the last deglaciation inferred from high-resolution pollen record in Gonghai Lake, Shanxi Province, China. *The Holocene* **27**, 835-846 (2017).

8. Xiao, J., Xu, Q., Nakamura, T., Yang, X., Liang, W. & Inouchi, Y. Holocene vegetation variation in the Daihai Lake region of north-central China: a direct indication of the Asian monsoon climatic history. *Quat. Sci. Rev.* **23**, 1669-1679 (2004).

9. Liu, Z., Wen, X., Brady, E. C., Otto-Bliesner, B., Yu, G., Lu, H. Y., Cheng, H., Wang, Y., Zheng, W., Ding, Y., Edwards, R. L., Cheng, J., Liu, W. & Yang, H. Chinese cave records and the East Asia Summer Monsoon. *Quat. Sci. Rev.* **83**, 115-128 (2014).

10. Zhang, H., Griffiths, M. L., Chiang, J. C. H., Kong, W. W., Wu, S., Atwood, A., Huang, J., Cheng, H., Ning, Y. & Xie, S. East Asian hydroclimate modulated by the position of the westerlies during Termination I. *Science* **362**, 580-583 (2018).

11. Chiang, J. C. H., Fung, I. Y., Wu, C., Cai, Y., Edman, J. P., Liu, Y., Day, J., Bhattacharya, T., Mondal, Y. & Labrousse, C. A. Role of seasonal transitions and westerly jets in East Asian paleoclimate. *Quat. Sci. Rev.* **108**, 111-129 (2015).
